# Association of Frailty with Intraoperative Complications in Older Patients Undergoing Elective Non-Cardiac Surgery

**DOI:** 10.3390/jcm14020593

**Published:** 2025-01-17

**Authors:** Mantana Saetang, Thitikan Kunapaisal, Sunisa Chatmongkolchart, Dararat Yongsata, Khwanrut Sukitpaneenit

**Affiliations:** Department of Anesthesiology, Faculty of Medicine, Prince of Songkla University, Hat-Yai 90110, Thailand; stmantana@gmail.com (M.S.); sunisasherry@gmail.com (S.C.); kidinnocent_tt@hotmail.com (D.Y.); khwanrut@gmail.com (K.S.)

**Keywords:** older patients, clinical frailty scale, modified frailty index-11, FRAIL scale, intraoperative complications

## Abstract

**Background:** Frailty is increasingly being recognized as a risk factor for adverse outcomes in older surgical patients undergoing surgery. We investigated the association between frailty and intraoperative complications using multiple frailty assessment tools in older patients undergoing elective intermediate- to high-risk non-cardiac surgery. **Methods:** This retrospective cohort study included 637 older patients scheduled for elective non-cardiac surgery. Frailty was assessed using the Clinical Frailty Scale (CFS), FRAIL scale, and modified Frailty Index-11 (mFI-11). The predictive ability of frailty tools was analyzed and compared using the area under the receiver operating characteristic curve (AUC). **Results:** Frailty was significantly associated with higher intraoperative complication rates (FRAIL scale: *p* = 0.01; mFI-11: *p* = 0.046). Patients considered frail using the mFI-11 were more likely to have unplanned intensive care unit admissions (*p* < 0.001). Those classified as frail by the FRAIL scale and mFI-11 had significantly higher rates of vasopressor/inotrope use (*p* = 0.001 and *p* = 0.005, respectively) and mechanical ventilation (*p* = 0.033 and *p* = 0.007, respectively). In the univariate analysis, frailty measured using the FRAIL scale was significantly associated with intraoperative complications (odds ratio [OR], 2.41; 95% confidence interval [CI]: 1.33–4.38; *p* = 0.004); this association was not significant in the multivariate analysis (adjusted OR, 1.69; 95% CI: 0.83–3.43; *p* = 0.148; AUC = 0.550). Atrial fibrillation, hemoglobin levels, anesthesia type, and surgical subspecialty were stronger predictors of intraoperative complications. **Conclusions:** Frailty assessments demonstrate the limited predictive ability for intraoperative complications. Specific comorbidities, surgical techniques, and anesthesia types play more critical roles. Comprehensive preoperative evaluations integrating frailty with broader risk stratification methods are necessary to enhance patient outcomes and ensure safety.

## 1. Introduction

The global population is aging, which has led to an increase in the growing number of older patients undergoing surgery. This demographic shift has significant implications for all areas of medicine, but it is particularly relevant in the peri-operative setting owing to age-related physiological changes and comorbidities. Advanced age is associated with a two- to four-fold increase in the rates of morbidity and mortality [1,2], and over 50% of patients who underwent major surgery are aged >65 years [3].

The prevalence of frailty increases exponentially with age. At 65 years of age, the prevalence is typically less than 10% but increases to over 50% in individuals aged >85 years [4]. Frailty is a multidimensional syndrome characterized by decreased reserves that leave an individual vulnerable to adverse outcomes because of decreased tolerance to stressors, including physical, physiological, or psychosocial stressors [5,6]. Surgery induces substantial physiologic stress even in healthy individuals [7]. Therefore, it is not surprising that the presence of frailty before surgery is strongly associated with an increased risk of adverse outcomes and higher resource utilization. Frailty is consistently associated with at least a two-fold increase in the risk of major morbidity, mortality, and readmissions [8,9].

Currently, evidence that frailty assessment occurs routinely in preoperative care remains scarce [10]. Many assessment models have been used to assess frailty. Based on available data, the Clinical Frailty Scale (CFS), modified Frailty Index-11 (mFI-11), and FRAIL scale can be used for less than a minute for the preoperative assessment. Assessing frailty in geriatric patients before surgery can aid in prognosis and optimization of care plans.

Older adults are at a higher risk of intraoperative complications due to age-related changes, such as reduced cardiovascular and respiratory reserves, altered pharmacokinetics, and increased comorbidities [11,12,13,14]. There is limited but growing evidence that frailty can predict intraoperative complications. Studies suggest that frail patients have a higher incidence of these complications, which can lead to poor postoperative outcomes [15,16,17]. Although frailty is well-established as a risk factor for postoperative complications, its impact on intraoperative outcomes remains underexplored.

This study aims to address this gap by evaluating the predictive value of frailty, as assessed using the CFS, mFI-11, and FRAIL scale, for intraoperative complications in older surgical patients undergoing surgery. Understanding this relationship will enhance preoperative risk stratification and potentially improve intraoperative management of this vulnerable population.

## 2. Materials and Methods

### 2.1. Study Design

This study is a retrospective cohort study that evaluated the association of frailty with intraoperative complications in older patients.

### 2.2. Study Setting

This study was conducted at a tertiary care center in southern Thailand, known for its specialized services in geriatric and surgical care. The data were extracted from electronic health records (EHRs) in Songklanagarind Hospital, Hat-Yai, Thailand from 20 August 2024 to 30 August 2024 after approval by the Institutional Ethics Committee of the Faculty of Medicine, Prince of Songkla University, Hat-Yai, Thailand.

### 2.3. Ethical Considerations

This study was conducted in accordance with the principles of the Declaration of Helsinki and was reviewed and approved by the Institutional Ethics Committee of the Faculty of Medicine, Prince of Songkla University, Hat-Yai, Thailand (approval reference: REC.67-309-8-1) on 17 August 2024. The need for informed consent was waived because of the retrospective nature of the cohort study.

### 2.4. Study Participants

The study included patients aged ≥60 years who were scheduled for elective non-cardiac surgery. Patients undergoing surgeries categorized as intermediate- to high-risk based on the cardiac risk stratification for non-cardiac procedures were eligible for inclusion. Only the first surgery performed during hospitalization was considered for each patient. The exclusion criteria were as follows: patients who were too unwell to undergo preoperative assessments and those receiving palliative care. Thus, 637 patients met the inclusion criteria and were included in the final analysis.

### 2.5. Ascertainment of Frailty

Frailty was assessed using three brief frailty assessment tools:

- CFS [18]: The CFS is a clinical tool that assesses frailty based on physical fitness and functional status. It classifies patients into nine categories, ranging from 1 (very fit) to 9 (terminally ill). In this study, patients were categorized as robust (scores 1–2), prefrail (scores 3–4), or frail (scores ≥ 5).
- mFI-11 [19]: An index derived from the accumulation of 11 deficits, including comorbidities and functional limitations, to measure frailty. Patients were categorized as robust (score = 0), prefrail (score < 0.27), or frail (score ≥ 0.27) based on their mFI-11 score.
- FRAIL Scale [20,21,22]: A phenotypic tool consisting of five items related to fatigue, resistance, ambulation, illness, and weight loss. A score of 0 indicates robustness, 1–2 indicates pre-frailty, and 3 or more indicates frailty.

### 2.6. Outcomes

The primary outcome was the association of the FRAIL scale, mFI-11, and CFS with intraoperative complications in older patients who underwent intermediate to high-risk surgery.

Intraoperative complications were defined as events requiring clinical intervention that occurred during surgery or in the immediate postoperative period in the recovery room, including transfusion of blood products (any of packed red blood cells, platelets, fresh frozen plasma, or cryoprecipitate), unplanned return to the operating room, unplanned admission to the intensive care unit (ICU), surgical complications (such as bowel perforation and periprosthetic fracture), new arrhythmia, hypotension requiring treatment, need vasopressor or inotrope, hypothermia (body temperature less than 35.5 °C), desaturation (SpO_2_ fall below 92%), massive blood loss (loss of ≥40% of total blood volume in a short period or blood loss exceeding 150 mL/min or 1.5 L within 1 h) and new onset delirium (acute and fluctuating disturbance in attention, awareness, and cognition). Complications were identified using EHR notes.

### 2.7. Data Source and Surgical Risk Classification

Data for this study were obtained from the EHR and included patient demographics, clinical assessments, and perioperative details. Intraoperative complications were identified using free-text EHR notes.

Surgeries were classified as intermediate- to high-risk based on cardiac risk stratification (Appendix A) following ESC Guidelines 2022 [23].

### 2.8. Statistical Analysis

Categorical variables are expressed as numbers and percentages; the differences between the groups were compared using the Chi-Square or Fisher’s exact tests, as appropriate. Continuous variables are expressed as means and standard deviations (SDs) or medians and interquartile ranges (IQRs); differences between groups were determined using the *t*-test or Rank-sum test, as appropriate. The Wilcoxon Rank-sum test was used to compare two independent samples. Logistic regression assessed associations between frailty classified by the FRAIL scale, mFI-11, and CFS with intraoperative complications. Variables included in the multivariate model were selected based on clinical relevance and significant associations observed in univariate analyses (*p* < 0.05). Cases with missing data for frailty or outcomes were excluded from the analysis. ROC curves were used to evaluate the predictive performance of frailty tools. Frailty was coded categorically as robust, prefrail, or frail. R-studio version 4.3.1 available at https://www.rstudio.com (accessed on 3 September 2024) was used for statistical analysis, and *p*-values < 0.05 indicated statistical significance.

## 3. Results

### 3.1. Study Population

In total, 637 patients met the inclusion criteria and were included in the final analysis. The overall intraoperative complication rate was 74.6% (475 of 637 patients). Frailty was associated with higher rates of certain intraoperative complications across different frailty scales (CFS, FRAIL scale, and mFI-11). The FRAIL scale and mFI-11 showed significant differences in intraoperative complications across frailty categories. However, the CFS did not show any statistically significant differences (Table 1). Notably, frail patients were more likely to experience unplanned admissions to the ICU and require blood product transfusions, vasopressor or inotropic support, and mechanical ventilation postoperatively compared to patients without frailty. All three frailty scales showed a statistically significant association between frailty and higher rates of blood product transfusion and intraoperative blood loss (*p* < 0.001). Only the mFI-11 showed a statistically significant association with unplanned ICU admission (*p* < 0.001). The FRAIL scale and mFI-11 showed significant associations with the need for vasopressors/inotropes and mechanical ventilation (Table 1).

### 3.2. Factors Associated with Intraoperative Complications

Several factors were significantly associated with intraoperative complications, whereas the Prognostic Nutritional Index (PNI) and metabolic equivalents (METs) < 4 were not associated with intraoperative complications (*p* = 0.345, *p* = 0.391, respectively) (Table 2). Multivariate analysis showed several independent risk factors for intraoperative complications, including increasing age (adjusted OR, 1.03; 95% CI: 1.00–1.06, *p* = 0.046), atrial fibrillation (adjusted OR, 9.24, 95% CI: 1.06–80.53, *p* = 0.044), and higher hemoglobin levels (adjusted OR, 0.80, 95% CI: 0.70–0.91, *p* < 0.001); certain surgical subspecialties (general surgeries were associated with the highest risk, whereas gynecological and urological surgeries were associated with lower risk), and the type of anesthesia (general anesthesia was associated with higher risk, whereas regional anesthesia and spinal anesthesia were associated with lower risk) (Table 3).

Frailty, as measured using the FRAIL scale, was significantly associated with increased odds of intraoperative complications in the univariate analysis (OR, 2.41, 95% CI: 1.33–4.38, *p* = 0.004). However, after adjusting for other variables in the multivariate analysis, this association was no longer statistically significant (adjusted OR, 1.69, 95% CI: 0.83–3.43, *p* = 0.148), with an AUC of 0.550 (Table 3). mFI-11 (OR 1.64, 95% CI 0.91–2.95, *p* = 0.101, AUC value of 0.525) and CFS (OR 1.08, 95% CI 0.75–1.57, *p* = 0.666, AUC value of 0.509) were not associated with intraoperative complications.

## 4. Discussion

This study highlights the association between frailty and intraoperative complications in older patients undergoing elective intermediate- to high-risk non-cardiac surgery using three frailty assessment tools: CFS, the FRAIL scale, and mFI-11. Frailty assessments, particularly those using the FRAIL scale and mFI-11, may provide valuable information for risk stratification. However, the multivariate analysis revealed poor discriminatory performance for all frailty tools. The relatively low AUC values for the frailty tools indicate their limited ability to independently predict intraoperative complications. This suggests that frailty assessments alone do not capture the complex interplay of patient- and procedure-specific factors influencing surgical outcomes.

The inconsistency between our findings and those of previous studies further emphasizes the complexity of this topic. For example, prior research has shown that frailty in patients, assessed using the mFI-11, was significantly associated with intraoperative complications (adjusted OR: 4.54, 95% CI, 1.18–17.60, *p* = 0.028) [24]. Similarly, Leopold-George et al. [25] found that frailty, assessed using the CFS, was associated with intraoperative complications. The discrepancy between our results and those of previous studies suggests that the relationship between frailty and intraoperative complications is more complex than previously understood. This may reflect the limited data currently available on this association. Additionally, differences in patient populations, surgical settings, and the application of frailty assessment tools may further contribute to these discrepancies.

In our study, the FRAIL scale showed a higher sensitivity, but lower specificity compared to the mFI-11, whereas the CFS had the lowest overall discriminatory ability. The differences in performance may reflect variations in the constructions measured by each tool. The CFS relies heavily on subjective clinical judgment [18], unlike the FRAIL scale and mFI-11, which focus on physical functioning (FRAIL scale) [20,21,22] and accumulated deficits (mFI-11) [19].

Additionally, several studies have reported that frail patients are more susceptible to intraoperative complications [9,24,25,26]. Our findings are consistent with those of previous studies in several respects. For instance, Leopold-George et al. [25] found that frailty was significantly associated with an increased incidence of blood transfusions and the need for vasopressors, which is consistent with our findings. However, we observed differences in other areas. Although several studies reported that frailty was significantly associated with an increased incidence of hypotension [25,26,27], our results showed that a high percentage of patients (78.4–84.7%) required treatment for hypotension, regardless of their frailty status. This suggests that, in our older population, the need for intraoperative blood pressure management was common across all levels of frailty, possibly because of factors such as diastolic heart failure and decreased venous compliance, which are common in older adults [28].

We found that frail patients, as assessed using mFI-11, had a significantly higher rate of unplanned ICU admissions, consistent with previous studies showing that frail patients who underwent comprehensive geriatric assessment had an increased likelihood of requiring high-level postoperative care [25,29] and predicting in-hospital mortality [30,31]. However, our study did not find significant differences in desaturation or intraoperative hypothermia based on frailty status, contrary to previous findings [25,32]. This may be because of the overall increased risk of hypothermia in geriatric patients, decreased muscle mass, and autonomic dysfunction [33].

Moreover, the findings suggest that intraoperative complications are more directly influenced by acute factors than by chronic vulnerabilities captured through frailty assessments. This may account for the observed variability in the predictive performance of frailty tools across different settings and contexts. Factors beyond frailty, such as specific comorbidities, surgical techniques, anesthesia type, and anesthesia management, appear to play critical roles in determining intraoperative complications in this patient population. Several independent risk factors were identified as significant contributors to intraoperative complications, including age, atrial fibrillation, hemoglobin levels, type of anesthesia, and surgical subspecialty. The results highlight the necessity of a comprehensive preoperative evaluation and risk stratification approach that integrates multiple factors, including but not limited to frailty status, to effectively optimize patient outcomes.

These factors align with findings from previous studies. Older patients are particularly vulnerable to intraoperative complications [34,35,36,37]. Intraoperative AF has been associated with prolonged ICU and hospital stays [38,39]. Preoperative anemia increases the need for red blood cell transfusions, further compounding risks during the perioperative period [40,41,42]. Regarding anesthesia type, recent evidence suggests that regional anesthesia does not significantly reduce 30-day postoperative mortality compared with general anesthesia in hip fracture surgery. However, regional anesthesia is associated with a lower incidence of intraoperative hypotension compared with general anesthesia [43]. Nevertheless, regional anesthesia has been linked to reduce in-hospital mortality. However, the type of anesthesia did not affect the occurrence of 30-day mortality, postoperative pneumonia, and delirium [44].

These findings highlight the multifactorial nature of intraoperative complications and the importance of a tailored, patient-cared approach to perioperative care. By addressing acute factors, such as anemia, atrial fibrillation, anesthesia type, and chronic vulnerabilities, such as frailty, clinicians can develop strategies to improve outcomes for older surgical patients.

Our findings support the integration of frailty assessments into routine preoperative evaluations, as emphasized by recent guidelines of the European Society of Anaesthesiology and Intensive Care [23]. These assessments should complement, rather than replace, other risk stratification methods to guide perioperative management and decision-making.

Despite the lack of significant associations of frailty tools in the multivariate analyses, our findings highlight the importance of comprehensive preoperative evaluation and risk stratification that considers multiple factors, including but not limited to frailty status. The complexity of intraoperative hemodynamics and complications in older patients highlights the need for vigilant monitoring and management of all patients in this age group, regardless of their frailty classification.

### Strengths and Limitations

This study adds to the growing evidence supporting the use of frailty assessments in surgical patients, specifically highlighting tools that are easy to implement and provide a meaningful predictive value. The use of multiple frailty tools allows for comprehensive analysis and comparison, providing insights into the most effective assessments for predicting intraoperative complications. Moreover, the findings underscore the potential role of preoperative frailty assessment in guiding perioperative management and risk stratification. However, the retrospective study design limits the ability to establish causal relationships because the observational nature precludes direct determination of the effects of frailty on intraoperative outcomes. Furthermore, the study was conducted at a single institution, which might limit the generalizability of the findings to other settings or populations. The exclusion of patients who were too unwell for preoperative assessments introduces a selection bias that may underestimate frailty-related risks as these individuals are likely to be more frail and at a higher risk of complications. Additionally, potential misclassification of outcomes or predictor variables, multiple comparisons testing, and risks of Type I and Type II errors further limit the robustness of the findings. Future research should focus on refining frailty assessments, integrating them into composite predictive models, and validating their effectiveness in diverse surgical contexts. A multidisciplinary, patient-centered approach that incorporates both chronic and acute factors is essential to optimize outcomes and improve the perioperative care of older surgical patients.

## 5. Conclusions

Frailty assessment tools demonstrated limited predictive performance for intraoperative complications. These findings suggest that other factors, including specific comorbidities, surgical techniques, and anesthesia type, play more critical roles in determining intraoperative risk compared to frailty alone. This underscores the need for a holistic approach to preoperative evaluation. Although frailty tools remain valuable for identifying vulnerable patients and guiding tailored interventions, their integration with broader risk stratification methods is essential to address the multifactorial nature of intraoperative complications and improve surgical outcomes and patient safety.

## Figures and Tables

**Table 1:** Comparison of intraoperative complications in terms of frailty status using CFS, the FRAIL Scale, and mFI-11 in older patients undergoing intermediate- to high-risk non-cardiac surgery.

Complications	Frailty Scale	Robust, *n* (%)	Prefrail, *n* (%)	Frail, *n* (%)	*p*-Value
Any intraoperative complications	CFS	25 (65.8)	265 (74.9)	185 (75.5)	0.433
	FRAIL	133 (73.1)	253 (71.9)	89 (86.4)	0.01
	mFI-11	156 (69.3)	251 (76.3)	68 (81.9)	0.046
Unplanned return to OR	CFS	0 (0)	0 (0)	1 (0.5)	0.466
	FRAIL	0 (0)	0 (0)	1 (1.1)	0.195
	mFI-11	0 (0)	0 (0)	1 (1.5)	0.146
Unplanned admission to the ICU	CFS	0 (0)	16 (6)	12 (6.5)	0.415
	FRAIL	4 (3)	15 (5.9)	9 (10.1)	0.086
	mFI-11	3 (1.9)	15 (6)	10 (14.7)	<0.001
Hypotension	CFS	22 (84.6)	215 (81.1)	153 (82.7)	0.854
	FRAIL	105 (78.4)	210 (83)	75 (84.3)	0.431
	mFI-11	133 (84.7)	201 (80.1)	56 (82.4)	0.494
New arrhythmia	CFS	0 (0)	2 (0.8)	0 (0)	0.562
	FRAIL	1 (0.7)	1 (0.4)	0 (0)	1
	mFI-11	1 (0.6)	1 (0.4)	0 (0)	1
Desaturation	CFS	1 (3.8)	16 (6)	14 (7.6)	0.691
	FRAIL	8 (6)	17 (6.7)	6 (6.7)	0.956
	mFI-11	7 (4.5)	18 (7.2)	6 (8.8)	0.394
Need inotrope	CFS	3 (11.5)	30 (11.3)	32 (17.3)	0.182
	FRAIL	6 (4.5)	43 (17)	16 (18)	0.001
	mFI-11	12 (7.6)	37 (14.7)	16 (23.5)	0.005
Hypothermia (<35.5 °C)	CFS	10 (38.5)	82 (30.9)	61 (33)	0.701
	FRAIL	46 (34.3)	81 (32)	26 (29.2)	0.724
	mFI-11	46 (29.3)	80 (31.9)	27 (39.7)	0.305
New onset delirium	CFS	0 (0)	2 (0.8)	4 (2.2)	0.455
	FRAIL	1 (0.7)	5 (2)	0 (0)	0.415
	mFI-11	1 (0.6)	4 (1.6)	1 (1.5)	0.735
On mechanical ventilator	CFS	0 (0)	9 (3.4)	14 (7.6)	0.063
	FRAIL	4 (3)	10 (4)	9 (10.1)	0.033
	mFI-11	3 (1.9)	12 (4.8)	8 (11.8)	0.007
Bradycardia	CFS	0 (0)	8 (3)	6 (3.2)	0.653
	FRAIL	3 (2.2)	8 (3.2)	3 (3.4)	0.866
	mFI-11	6 (3.8)	8 (3.2)	0 (0)	0.336
Surgical complication	CFS	1 (3.8)	14 (5.3)	10 (5.4)	0.945
	FRAIL	7 (5.2)	14 (5.5)	4 (4.5)	0.931
	mFI-11	6 (3.8)	13 (5.2)	6 (8.8)	0.303
Massive blood loss	CFS	0 (0)	2 (0.8)	6 (3.2)	0.144
	FRAIL	1 (0.7)	4 (1.6)	3 (3.4)	0.291
	mFI-11	0 (0)	6 (2.4)	2 (2.9)	0.076
Blood transfusion	CFS	6 (23.1)	49 (18.5)	69 (37.3)	<0.001
	FRAIL	23 (17.2)	64 (25.3)	37 (41.6)	<0.001
	mFI-11	28 (17.8)	67 (26.7)	29 (42.6)	<0.001
Durations of sx. (mins); median (IQR)	CFS	267.5 (183.8–340)	250 (175–373.8)	260 (180–350)	0.975
	FRAIL	247.5 (180–350)	255 (170–360)	265 (190–360)	0.482
	mFI-11	270 (175–360)	240 (180–360)	265 (182.5–342.5)	0.688
Blood loss (mL); median (IQR)	CFS	50 (20–287.5)	100 (20–300)	150 (50–400)	0.003
	FRAIL	50 (20–250)	100 (27.5–400)	150 (50–475)	<0.001
	mFI-11	100 (20–300)	100 (20–300)	200 (40–425)	0.048

CFS = Clinical Frailty Scale; ICU = intensive care unit; IQR = interquartile range; mFI-11, modified Frailty Index-11; OR = operating room; sx = surgery; mL = milliliters.

**Table 2:** Factors associated with intraoperative complications in older patients undergoing intermediate- to high-risk non-cardiac surgery.

Factors Associated with Intraoperative Complications	Intraoperative Complications		*p*-Value
	No (*n* = 162)	Yes (*n* = 475)	
Age (years); median (IQR)	68 (64–74)	70 (65–76)	0.005
Sex: male, *n* (%)	56 (34.6)	250 (52.9)	<0.001
BMI; median (IQR)	25.3 (22.4–28.4)	23.3 (20.8–26.6)	<0.001
Comorbidity, *n* (%)	145 (89.5)	451 (94.9)	0.024
Ischemic heart disease	9 (5.6)	54 (11.4)	0.047
Hypertension	91 (56.2)	303 (63.8)	0.103
Dyslipidemia	93 (57.4)	281 (59.2)	0.765
Diabetes	29 (17.9)	99 (20.8)	0.488
Chronic lung disease	5 (3.1)	34 (7.2)	0.094
CKD stage ≥ 3	11 (6.8)	61 (12.8)	0.05
CVD (previous stroke or TIA)	9 (5.6)	47 (9.9)	0.128
PAD	5 (3.4)	16 (3.5)	1
Cancer	52 (32.1)	215 (45.3)	0.005
Metastasis	15 (9.3)	91 (19.2)	0.005
AF	1 (0.6)	20 (4.2)	0.05
ASA classification score; median (IQR)	2 (2–3)	3 (2–3)	<0.001
Surgical subspecialty, *n* (%)			<0.001
General surgery	24 (14.8)	162 (34.1)	
Orthopedic surgery	75 (46.3)	96 (20.2)	
Vascular surgery	16 (9.9)	89 (18.7)	
Gynecologic surgery	20 (12.3)	34 (7.2)	
Thoracic surgery	8 (4.9)	47 (9.9)	
Urologic surgery	19 (11.7)	47 (9.9)	
Cardiac risk stratification surgical procedure, *n* (%)			<0.001
High-risk	22 (13.6)	150 (31.7)	
Intermediate-risk	140 (86.4)	323 (68.3)	
Hb; median (IQR)	12.5 (11.6–13.3)	11.8 (10.6–13)	<0.001
PNI; median (IQR)	47.5 (44– 52)	48 (43– 52)	0.345
Choice of anesthesia, *n* (%)			<0.001
GA	58 (35.8)	271 (57.2)	
RA	4 (2.5)	7 (1.5)	
Combined GA + RA	27 (16.7)	145 (30.6)	
SB	7 (4.3)	8 (1.7)	
SB + RA	66 (40.7)	43 (9.1)	
METs			0.391
<4	19 (22.1)	68 (17.4)	
≥4	67 (77.9)	322 (82.6)	
Frail by FRAIL scale, *n* (%)	14 (8.6)	89 (18.7)	0.004
Frail by mFI-11, *n* (%)	15 (9.3)	68 (14.3)	0.13
Frail by CFS, *n* (%)	60 (37)	185 (38.9)	0.735

AF = atrial fibrillation; ASA = American Society of Anesthesiologists; BMI = Body Mass Index; CKD = chronic kidney disease; CFS = Clinical Frailty Scale; CVD = cerebrovascular disease; GA = general anesthesia; Hb = hemoglobin level; PNI = prognostic nutritional index; IQR = interquartile range; METs = metabolic equivalents; mFI-11 = modified Frailty Index-11; PAD = peripheral arterial disease; RA = regional anesthesia; SB = subarachnoid block; TIA = transient ischemic attack.

**Table 3:** Univariate and multivariate analysis of factors associated with intraoperative complications in older patients undergoing intermediate- to high-risk non-cardiac surgery.

Factors Associated with Perioperative Complications	Univariate Analysis		Multivariate Analysis	
	OR (95% CI)	*p*-Value	OR (95% CI)	*p*-Value
Age	1.04 (1.01–1.06)	0.004	1.03 (1.00–1.06)	0.046
Sex: female vs. male	0.47 (0.33–0.69)	<0.001	0.61 (0.37–1.01)	0.056
AF: yes vs. no	7.19 (0.96–53.98)	0.055	9.24 (1.06–80.53)	0.044
subspecies: ref. = General surgery				
Orthopedic surgery	0.19 (0.11–0.32)	<0.001	1.09 (0.47–2.52)	0.849
Vascular surgery	0.82 (0.41–1.62)	0.569	0.69 (0.32–1.51)	0.352
Gynecologic surgery	0.25 (0.13–0.51)	<0.001	0.27 (0.12–0.6)	0.001
Thoracic surgery	0.82 (0.34–1.95)	0.653	0.95 (0.38–2.42)	0.921
Urologic surgery	0.35 (0.18–0.7)	0.003	0.39 (0.18–0.83)	0.014
Hb	0.81 (0.72–0.9)	<0.001	0.80 (0.70–0.91)	<0.001
Choice of anesthesia: ref. = GA				
RA	0.38 (0.11–1.33)	0.128	0.2 (0.05–0.85)	0.029
combine GA + RA	1.11 (0.68–1.84)	0.673	1.51 (0.83–2.75)	0.181
SB	0.25 (0.09–0.7)	0.009	0.21 (0.07–0.65)	0.007
SB + RA	0.14 (0.09–0.23)	<0.001	0.11 (0.05–0.24)	<0.001
Frail by FRAIL scale	2.41 (1.33–4.38)	0.004	1.69 (0.83–3.43)	0.148
Frail by mFI-11	1.64 (0.91– 2.95)	0.101		
Frail by CFS	1.08 (0.75– 1.57)	0.666		

AF = atrial fibrillation; CFS = Clinical Frailty Scale; CI = confidence interval; GA = general anesthesia; Hb = hemoglobin level; mFI-11 = modified Frailty Index-11; OR = odds ratio; RA = regional anesthesia; SB = subarachnoid block.

## Data Availability

All data generated or analyzed during this study are included in this published article.

## Abbreviations

The following abbreviations are used in this manuscript:

CFS	Clinical Frailty Scale
CI	Confidence interval
EHR	Electronic health record
ICU	Intensive care unit
PNI	Prognostic Nutritional Index
SD	Standard deviation
AUC	Area under the receiver operating characteristic curve
OR	Odds ratio
mFI-11	Modified Frailty Index-11

## Appendix A

**Table A1:** Surgical Risk Estimates According to Type of Surgery or Intervention.

Low Surgical Risk (<1%)	Intermediate Surgical Risk (1–5%)	High Surgical Risk (>5%)
Breast	Carotid asymptomatic (CEA)	Adrenal resection
Dental	Carotid symptomatic (CEA)	Aortic and major vascular artery
Eye	Endovascular aortic aneurysm repair	Carotid symptomatic (CAS)
Endocrine: Thyroid	Head or neck surgery	Duodenal-pancreatic surgery
Gynecological: minor	Intraperitoneal: splenectomy, hiatal hernia	Liver resection, bile duct surgery
Orthopedic: minor (meniscectomy)	Intrathoracic non-major	Esophagectomy
Reconstructive	Neurological or orthopedic major	Open lower limb revascularization
Superficial surgery	Peripheral arterial angioplasty	Pneumonectomy
Transurethral resection of prostate	Renal transplant	Pulmonary or liver transplant
VATS minor lung resection	Urological or Gynecological major	Repair of perforated bowel
		Total cystectomy

VATS = Video-assisted thoracic surgery, CEA = Carotid endarterectomy, CAS = Carotid artery stent.
